# The Role of Nicotinic Receptors on Ca^2+^ Signaling in Bovine Chromaffin Cells

**DOI:** 10.3390/cimb46010052

**Published:** 2024-01-17

**Authors:** Amparo Gil, Virginia González-Vélez, Luis Miguel Gutiérrez, José Villanueva

**Affiliations:** 1 Departamento de Matemática Aplicada y CC de la Computación, Universidad de Cantabria, 39005 Santander, Spain; amparo.gil@unican.es; 2 Departamento Ciencias Básicas, Universidad Autónoma Metropolitana Azcapotzalco, Mexico City 02128, Mexico; 3 Instituto de Neurociencias, CSIC-Universidad Miguel Hernández. Ctra de Valencia S/N, Sant Joan d’Alacant, 03550 Alicante, Spain; luisguti@umh.es

**Keywords:** Ca^2+^, acetylcholine nicotinic receptors, chromaffin cells, exocytosis

## Abstract

Chromaffin cells have been used as a physiological model to understand neurosecretion in mammals for many years. Nicotinic receptors located in the cells’ membrane are stimulated by acetylcholine, and they participate in the exocytosis of chromaffin granules, releasing catecholamines in response to stress. In this work, we discuss how the participation of nicotinic receptors and the localization of active zones in the borders of the cytoskeleton can generate local calcium signals leading to secretion. We use a computational model of a cytoskeleton cage to simulate Ca^2+^ levels in response to voltage and acetylcholine pulses. We find that nicotinic receptors are able to enhance the differences between local and average calcium values, as well as the heterogeneous distributions around the active zones, producing a non-linear, highly localized Ca^2+^ entry that, although consisting of a few ions, is able to improve secretion responses in chromaffin cells. Our findings emphasize the intricate interplay among nicotinic receptors, the cytoskeleton, and active zones within chromaffin cells as an example of Ca^2+^-dependent neurosecretion in mammals.

## 1. Introduction

The medulla of the adrenal glands positioned above the kidneys consists primarily of chromaffin cells. These specialized cells are involved in the body’s response to fear, stress, or conflict, known as the “fight or flight” response. This response is possible thanks to the release of catecholamines from chromaffin cells into the bloodstream. These hormones help prepare our body for action, enhancing physical performance and increasing alertness. In chromaffin cells, as well as in other neuroendocrine cells, the ion Ca^2+^ manages the process of hormone secretion [1]. 

Acetylcholine (ACh), the neurotransmitter released from the sympathetic nervous system, specifically stimulates the nicotinic receptors (nAChRs) located in the membrane of chromaffin cells to trigger catecholamine secretion. Different subtypes of nAChRs activate a wide variety of physiological functions, and those expressed in neurons have been related to important neurological or behavioral disorders, such as nicotine dependence and Alzheimer’s or Parkinson’s disease, thus becoming a relevant potential target for treatments [2].

The main subtype of nAChRs that are present in chromaffin is a pentamer assembly of α3 and β4 subunits. These receptors, once opened by an ACh pulse, increase the permeability of Na^+^, K^+^, and Ca^2+^. Although the existence of homomeric α7 receptors has also been evidenced, it is also considered that they do not contribute to the secretion of catecholamines [3]. On the other hand, it has been described that heteromeric receptors α3β4 are desensitized more slowly (<20 s) than α7 (<1 s), so that in tests of stimuli of several seconds with ACh, the contribution to the increased permeability of α3β4 receptors would seem to be more decisive [4]. Recently, using α-bungarotoxin and α-conotoxin labeled with the appropriate fluorophores using the FRET technique (Förster resonance energy transfer), the existence of a true interaction between the subtypes of α3β4 and α7 receptors in human chromaffin cells was evidenced. Moreover, under repeated ACh stimuli, such an interaction would enhance the activity of both receptors, preventing their desensitization [5].

In resting conditions (no-stress situation), small and non-frequent pulses of ACh stimulate the nAChRs, which generate a small Ca^2+^ current leading to a low, basal hormone secretion. In contrast, under stress, high rates of ACh stimulate chromaffin cells to reach high secretion rates. This latter process is possible thanks to the recruitment of voltage-dependent calcium channels (VDCCs) that allow a much higher Ca^2+^ current that consequently triggers rapid vesicle fusion and release. A two-step model was proposed to explain these effects of ACh: (1) the low entry of Ca^2+^ through nAChRs serves mainly to keep the secretory machine loaded with vesicles; and (2) in this way, the cell is primed to respond with explosive catecholamine secretion upon depolarization and the rapid entry of Ca^2+^ via VDCC [6]. But what could be the importance of the small current managed by nAChRs? As postulated in this last reference, these few calcium ions may help vesicles to move towards the active zones of secretion to be prepared for stress situations. Then, it is also interesting to analyze the contribution of these receptors to the Ca^2+^ signals leading to exocytosis in chromaffin cells. 

The precise control of Ca^2+^ signals within chromaffin cells is crucial for the regulation of exocytosis. Ca^2+^ acts as a key mediator that coordinates the timing and efficiency of hormone release. Calcium ions bind to specific proteins, such as SNARE proteins, to trigger a cascade of events that culminate in vesicle fusion and hormone secretion. Understanding the intricate interplay between Ca^2+^ signaling and exocytosis in chromaffin cells is of great importance since they are excellent models to understand neurosecretion [7]. Moreover, this regulation of catecholamine release could have significant implications for many physiological responses and contributes to various health problems such as cardiovascular disorders, essential hypertension, and neurodegenerative and stress-related diseases [8,9].

The cytoskeleton in chromaffin cells is a dynamic structure that plays a key role in Ca^2+^ spatial signals and secretion [10]. It has been observed that Ca^2+^ is mainly distributed in the empty spaces (cages) of the cytoskeleton, allowing a heterogeneous spatial distribution of this ion. Moreover, VDCCs are strategically positioned in the borders of cytoskeletal cages. These cytoskeletal cages consist of a network of protein filaments that provide structural support and organization to the cell. Interestingly, VDCCs are found in close proximity to the secretory machinery, which includes vesicles and SNARE proteins. In a previous study, we found that the co-localization of VDCCs and the secretory machinery in the borders of the actin cytoskeleton can improve the efficiency of the exocytotic process [11].

In [12], initial evidence that a population of α3β4 nAChR clusters are located very close to active secretory sites formed by SNARE proteins was provided. In the present work, we provide further experimental evidence indicating that nicotinic receptors seem to be in close proximity not only to the cortical actin, but also to the exocytotic machinery. We then utilize a computational model incorporating these spatial characteristics as well as the kinetic aspects observed in previous experiments in order to analyze the calcium signaling that leads to exocytosis in bovine chromaffin cells. We show how the participation and localization of nicotinic receptors close to the secretory machinery can reproduce the spatial heterogeneity of calcium signals, and that this ensemble creates a synergy that improves the efficiency of the secretory response. 

## 2. Materials and Methods

### 2.1. Experimental Methods and Materials

#### 2.1.1. α3-nAchR, SNAP-25, and LifeAct DNA Constructs

The complementary DNA corresponding to the human α3-nAchR neuronal subunit [13] was cloned into the appropriate site of the pEGFP-C3 expression vector (Clontech, Palo Alto, CA, USA) to express this protein in the frame C-terminal to EGFP (construct α3-EGFP). The pDsRed-SNAP25 constructs (SNAP25-DsRed) were obtained from cDNA corresponding to the SNAP25a isoform [14] cloned in a similar way into the appropriate site of the pDsRed-C3 expression vector (Clontech, Palo Alto, CA, USA).

The pCMV–LifeAct^®^–TagRFP (LifeAct-RFP) constructs were obtained from Ibidy^®^ (Ibidy GmbH, Gräfelfing, Germany).

#### 2.1.2. Chromaffin Cell Culture and Electroporation

Chromaffin cells were isolated from bovine adrenal glands via collagenase digestion and were further separated from the debris by centrifugation on Percoll gradients as described elsewhere [15]. For electroporation, we used Amaxa’s nucleofector II^®^ system (Lonza, ref AAD-1001, Cologne, Germany) with the primary mammalian neuronal cells kit according to the manufacturer’s instructions (Lonza, red VVPG-1001, Cologne, Germany) (Program O-005, Amaxa GmbH, Cologne, Germany).

After transfection, the cells were maintained as monolayer cultures in Dulbecco’s modified Eagle’s medium (DMEM) supplemented with 10% fetal calf serum, 10 µM cytosine arabinoside, 10 µM 5-fluoro-2′-deoxyuridine, 50 U/mL penicillin, and 50 µg/mL streptomycin. The cells were harvested at a density of 150,000 cells/cm^2^ in 22 mm diameter coverslips coated with poly-lysine. The fluorescence of the cells was studied 24 to 48 h post plating. 

#### 2.1.3. Confocal Microscopy Studies of Fluorescent Structures 

Fluorescence was visualized with an Olympus Fluoview FV300 confocal laser system mounted on an IX-71 inverted microscope incorporating a 100× UPlanSApo oil immersion objective. We used the sequential mode to register the distinct signals with a 488 nm argon ion 40 mW to excite EGFP and a 543 nm He/Ne 10 mW for DsRed or RFP (both lasers from Melles Griot, Carlsbad, CA, USA).

Using confocal microscopy, the location of the expression of the constructs was analyzed after 24 to 48 h after transfection. These expressions were analyzed for single α3-EGFP transfection or for a double transfection of α3-EGFP with other fluorescent constructs expressing proteins involved in the secretory machinery (LifeAct-RFP or SNAP25-DsRed). 

Typically, confocal microscopy planes were obtained from the upper external (TOP) or lower (BOTTOM) area of each individual cell in order to perform quantitative measurements in the area of the membrane. The records acquired in the central plane of the cell (sagittal) were only for qualitative purposes as visual confirmation of the apparent existence of a peripheral label in the cells. 

#### 2.1.4. Image Analysis

Image analysis was performed using the free software FIJI ImageJ 1.52i. Each individual image of two channels was analyzed to measure the distances between the centroids of the different structures identified in order to compare their relative distribution. When we wanted to analyze the colocalization and overlapping of the pixels between individual nearby structures, the ImageJ JACoP complement was used [16], obtaining the Pearson and Manders coefficients between such structures for each region of interest (ROI) studied. In all cases, the data were subsequently processed with the GraphPad Prism 5.01 software and the graphics and images were optimized with Adobe Photoshop CS5, v.12.0.4.

### 2.2. Geometrical Model

**Figure 1:**
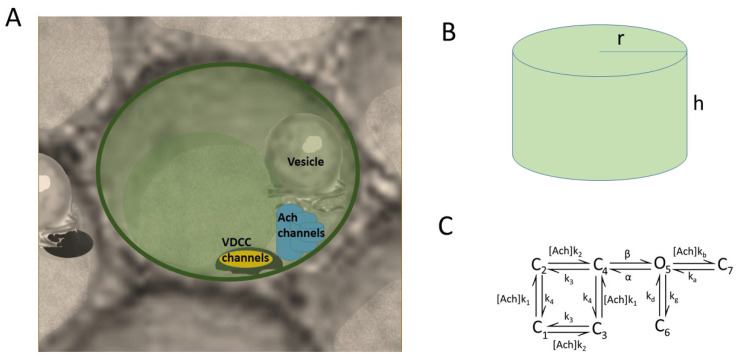
Geometrical model. (**A**) First slice of the cylinder shown in (**B**) where VDCC, ACh receptors, and vesicles are located. This draw exemplifies the Border configuration tested in the work where all mechanisms are located next to the perimeter of the slice. (**B**) Cylinder representing a prototypical cytoskeleton cage with a radius (r) of 0.3 μm and a height (h) of 1 μm. (**C**) State model of the nicotinic receptors.

We propose a geometrical model resembling a prototypical cytoskeletal cage where the voltage-dependent calcium channels (VDCCs), vesicles, secretory machinery, and nicotinic receptors are present (Figure 1). Figure 1A shows the region corresponding to the cell membrane where a cluster of three VDCCs, a cluster of three nicotinic receptors, and one SNARE microdomain and its associated vesicle are all located in the cytoskeletal cage. In particular, in this draw, we show the Border configuration for the molecular elements. Figure 1B depicts the cylindrical domain used to represent the prototypical cytoskeletal cage, which has a radius (r) of 0.3 μm, according to the average radius (0.18–0.24 μm) found for exocytotic events in chromaffin cells [17], and a height (h) of 1 μm. The height of the cylinder is divided into slices of 30 nm to quantify the calcium concentrations over time in these regions.

In our model, we consider that the cluster of VDCCs is formed by two P/Q- and one L-type calcium channel, in light of the experimental findings concerning the involvement of high-voltage-activated calcium channels in the exocytotic response of bovine chromaffin cells [1,18,19]. Both P/Q- and L-type calcium channel subtypes were modeled as three-state Markov processes, each characterized by distinct transition rates tailored to match their specific current-to-voltage profiles. Once the VDCCs and/or nicotinic receptors are open, calcium ions enter the domain, and then buffered diffusion and exocytosis are simulated using our particle-based mathematical program implemented in Fortran, which has already been proven to be successful in the study of calcium dynamics and secretion in active zones of neuroendocrine cells [20]. The calcium permeability through nicotinic receptors is calculated with a seven-state model (Figure 1C) previously described in [12]. All dynamic plots and calcium maps are the average of ten simulations, since our software is based on a Monte Carlo method. 

## 3. Results

### 3.1. Experimental Findings

#### 3.1.1. Localization of α3β4 nAChRs Structures Close to Actin Cytoskeleton 

**Figure 2:**
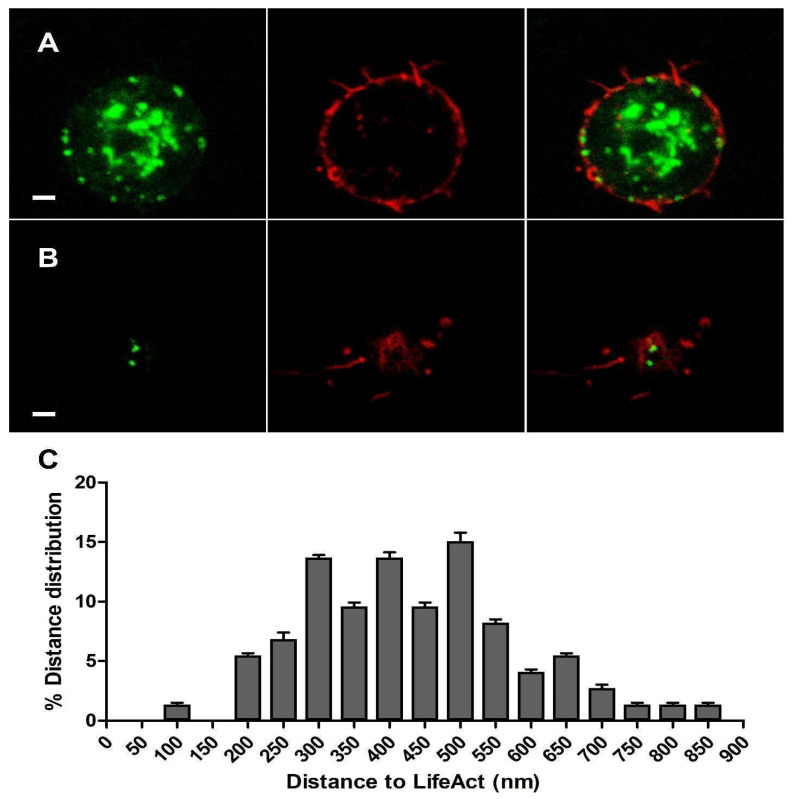
Plasmalemmal α3-GFP (green) and LifeAct-RFP (red) co-expression in cultured chromaffin cells. Panel (**A**) shows images corresponding to a confocal microscopy plane obtained from the central zone of the cell. Panel (**B**) corresponds to a polar upper section (TOP) image of the cell. In both planes, the left image corresponds to the α3-EGFP channel, the central image to the LifeAct-RFP channel, and the right image to both merged channels. Panel (**C**) shows the XY distance distribution (nm) between the α3-EGFP and LifeAct-RFP centroids obtained from cortical plane images at the top or bottom of the cells (*n* = 86, 9 cells from 2 distinct cultures). Bar: 1 μm.

We co-expressed α3-EGFP and LifeAct-RFP constructs in cultured bovine chromaffin cells. After 48 h incubation, we performed a sequential protocol of excitation and acquisition (see Section 2). α3-EGFP fluorescence could be seen to form some patches near the fluorescence of LifeAct-RFP, which were especially dense in the F-actin cortical barrier as expected in chromaffin cells [21] (Figure 2A). This distribution confirmed that the α3-EGFP expressed subunits, and probably their resulting heteromeric α3β4 nAChRs structures, were located very close to the F-actin cortical barrier.

Each individual cortical plane image at the top or bottom of the cells was analyzed using ImageJ software v.1.49p to measure the distances between the centroid of each expressed α3-EGFP subunit and the centroid corresponding to the nearest expressed LifeAct-RFP in order to compare their relative distribution (Figure 2B). The distribution of such distances in the cortical layer was analyzed (*n* = 86, nine cells from two distinct cultures), showing a population of α3-EGFP-expressed subunits that localized very close to the expressed LifeAct-RFP clusters with an average distance of 447.8 ± 45.9 nm (Figure 2C).

#### 3.1.2. Localization of α3β4 nAChRs in the Vicinity of Secretory Machinery

To confirm that such heteromeric α3β4 nAChRs are distributed near the vicinity of the secretory machinery, we performed a double transfection with α3-EGFP and SNAP25-DsREd. After 48 h incubation, we visualized the samples using the sequential protocol of excitation and acquisition described in Section 2.

**Figure 3:**
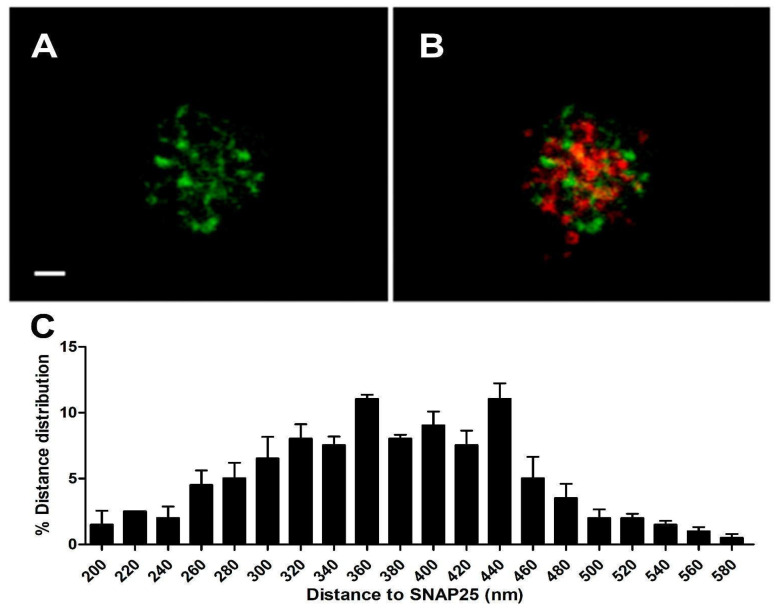
Cortical distribution of α3-EGFP and SNAP25-DsRed 48 h co-expression. Panel (**A**) shows the α3-EGFP channel alone. Panel (**B**) shows a merged image of α3-EGFP (green) and SNAP25-Dsred (red) co-expression. Panel (**C**) shows the average distance distribution between the centroids of each α3-EGFP patch and the centroids of the nearest SNAP-25-DsRed (see text). (*n* = 95, 12 cells, from 2 distinct cultures.) All records were obtained using sequential laser excitation and acquisition. Bar: 1 μm.

We employed ImageJ software v. 1.49p and followed a similar procedure to the one described in Section 2.1.1 to analyze the distance between the centroid of each expressed α3-EGFP subunit and the centroid corresponding to the nearest expressed SNAP25-DsRed (Figure 3). The distance distribution of such α3-EGFP structures in the cortical layer was analyzed (*n* = 95, 12 cells from two distinct cultures), showing a population of α3-EGFP expressed subunits that localized very close to the expressed SNAP25-DsRed clusters with an average distance of 380.8 ± 6.2 nm (Figure 3C).

The co-expression of SNAP25-DsRed and α3-EGFP constructs, using the same incubation conditions, resulted in an α3-EGFP population distributed very close to the characteristic cortical SNAP25-DsRed location (Figure 3B). Taking all of these data into account, we were sure that a population of α3-EGFP-expressed proteins seemed to be near not only the cortical actin, but also the exocytotic machinery (SNAP25). The mean distance between the SNAP25-DsRed centroid and α3-EGFP subunits was equal to 380.8 ± 6.2 nm. This result is in agreement with the mean distance of SNAP25-Dsred vs. α3-Ab given in [12].

### 3.2. Modeling

Previous work has reported non-uniform spatial patterns due to the participation of the actin cytoskeleton in stimulated chromaffin cells [10], as well as the contribution of the heteromeric α3β4 nAChR structures to the exocytotic process [12]. In this section, we consider a comprehensive computational model (described in Section 2) that incorporates nAChRs, VDCCs, and the secretory machinery inside a prototype cytoskeleton cage to quantify the contribution of these receptors in the calcium signaling leading to secretion in bovine chromaffin cells. 

#### 3.2.1. Localization of Voltage-Dependent Calcium Channels (VDCCs) Modulate the Intensity and Distribution of Calcium Signals

**Figure 4:**
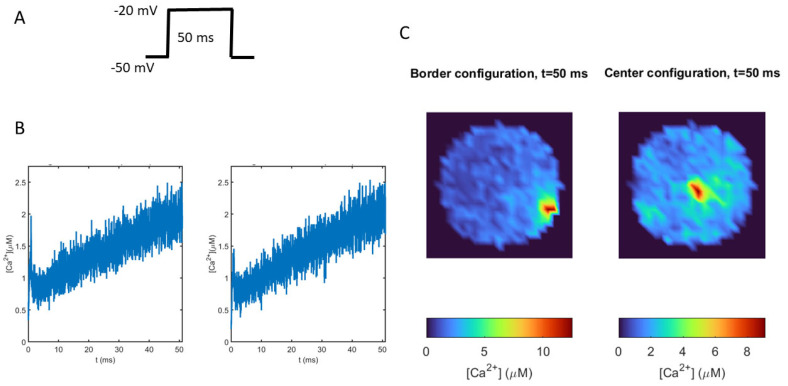
Differences in calcium distributions and intensities when VDCCs are located in different zones of the cytoskeletal cage. (**A**): The 50 ms depolarizing pulse from −50 mv to −20 mV used to stimulate VDCC in the model. (**B**): Dynamics of average calcium obtained when VDCCs are located on the border (left panel) or in the center (right panel) of the cage model. (**C**): Calcium maps showing the spatial distribution of calcium ions, for the two locations, when the voltage pulse had finished (after 50 ms).

To test the influence of VDCC localization on calcium signals, we compared the intracellular calcium spatial distributions obtained for two different scenarios: one in which the cluster of VDCCs, nAChRs, and the secretory machinery is located on the border of the cytoskeleton cage (Border configuration), and another where these molecular elements are distributed inside the cage (Center configuration). A 50 ms depolarizing pulse was used to stimulate the VDCCs (Figure 4A). As shown in Figure 4B, the dynamics of the average calcium are very similar for both spatial localizations, while the local calcium values are completely different (Figure 4C). After 50 ms, that is, when the voltage pulse had finished, the Border configuration induced a polarized calcium distribution with a peak value in the area where the channels and the secretion machinery were located, while the Center configuration generated a more homogeneous distribution. Notice that the calcium peak value when the VDCCs are on the border was 33% greater than when the VDCCs were in the center of the cage. These results show that clusters of VDCCs, together with the confinement due to the cytoskeleton cage, are able to induce high local calcium signals that cannot be appreciated in the average calcium dynamics using whole-cell models.

#### 3.2.2. Nicotinic Receptors Reinforce the Border Effect of the Cytoskeletal Cage on Calcium Concentrations

**Figure 5:**
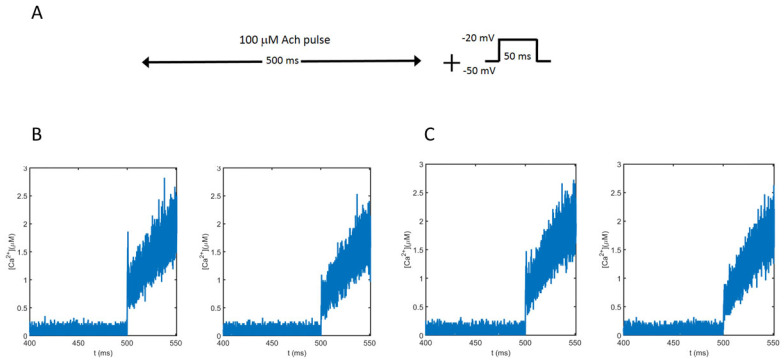
Average calcium concentrations in response to the simulated protocol shown in (**A**). Values obtained between 0–30 nm (left panels in (**B**,**C**)) and 60–90 nm (right panels in (**B**,**C**)) from the cell membrane. (**B**): Results obtained for the Border configuration. (**C**): Results obtained for the Center configuration.

To test the influence of nicotinic receptors in the calcium signal developed by electrical and chemical stimulation, we simulated the submembrane calcium concentrations obtained in response to a 100 μM ACh pulse lasting 500 ms followed by a brief (50 ms) depolarizing pulse from −50 mV to −20 mV (Figure 5A), in accordance with the experimental protocols used to study nAChRs and exocytosis [6]. The ACh pulse stimulates the receptors while the depolarizing pulse partially opens the VDCCs. The dynamics of the averaged calcium concentrations obtained for the two geometrical configurations (Border vs. Center) at two different distances from the cell membrane (0–30 nm and 60–90 nm) were obtained. Figure 5B shows the results obtained for the Border configuration, and Figure 5C shows the results for the Center configuration. As can be seen, all average calcium concentrations are very similar, and before 500 ms, the calcium entering into the cell in response to the ACh pulse is very low, as expected. However, after this time, when the voltage pulse appears, there is a clear increase in the intracellular calcium concentrations at both distances, and in both configurations, due to the activation of the VDCCs. 

**Figure 6:**
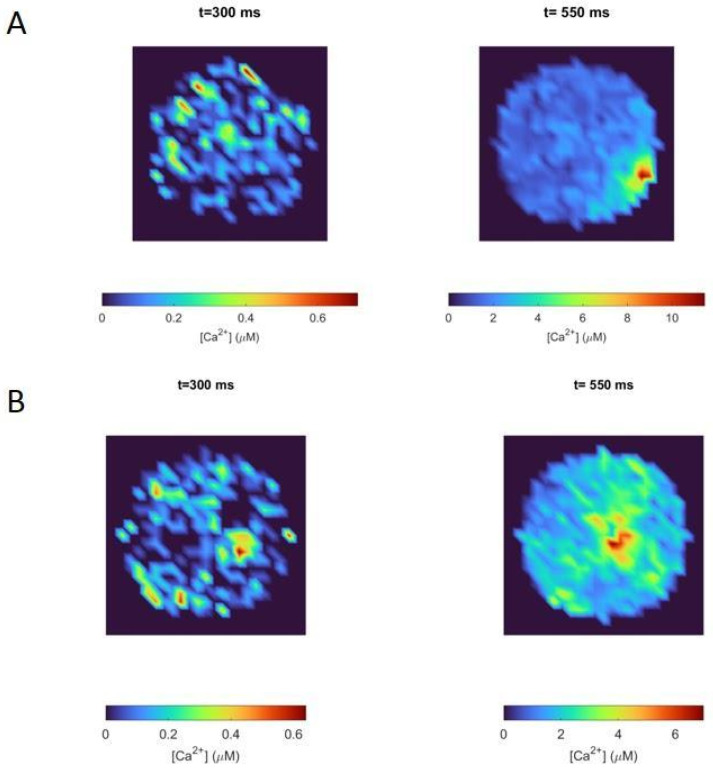
Calcium maps showing local calcium concentrations up to 30 nm from the cell membrane, at two different times after starting the simulated protocol shown in Figure 4A. (**A**): Border configuration. (**B**): Center configuration.

Two-dimensional calcium maps showing the corresponding local calcium concentrations in the region between 0 and 30 nm from the cell membrane, at specific times, are shown in Figure 6. These maps represent how calcium is distributed in this region of the cell in response to the same stimulation protocol used for Figure 5. We chose two specific times (300 ms and 550 ms) to make clear the influence of nicotinic receptors in local calcium signals. These two time values were chosen to represent the calcium distribution during the ACh prepulse period (t = 300 ms) and when the depolarizing pulse had finished (t = 550 ms). As observed in Figure 6, the Border configuration (Figure 6A) induced a polarized distribution of calcium and allowed the cell to reach higher values at 550 ms than the Center configuration (Figure 6B). Indeed, the peak value of calcium, at the end of the pulse, for the Border configuration was about 40% higher than the one for the Center configuration. Since the border localization of nicotinic receptors and VDCCs was the actual localization observed in experiments [12], Figure 4C and Figure 6A exhibit the real participation of nicotinic receptors in the calcium signals developed by a stimulated chromaffin cell.

Although the calcium ions entering through the nicotinic receptors were scarce (as seen in Figure 5B,C before 500 ms), the non-linear nature of this process together with the border effect caused an increase in the polarization of the calcium signal compared to the situation in which there was no prior calcium entry through the nicotinic receptors (Figure 4C versus Figure 6A,B at 500 ms). Therefore, Figure 6A corresponds to the local calcium signal developed thanks to the co-localization of the nicotinic receptors, VDCCs, and secretory machinery on the borders of the actin cytoskeleton, which is the geometrical arrangement reached after ACh stimulates a chromaffin cell, as discussed in [6,10].

#### 3.2.3. Secretion Is Improved Thanks to Local Ca^2+^ Signals

As described in previous sections, the participation of nicotinic receptors in calcium signals is clear even though they allow few calcium ions to enter the cell once they are activated by ACh. Moreover, as we previously observed [12], these receptors are located close to the secretory machinery, so it is also interesting to analyze their contribution to secretion.

**Figure 7:**
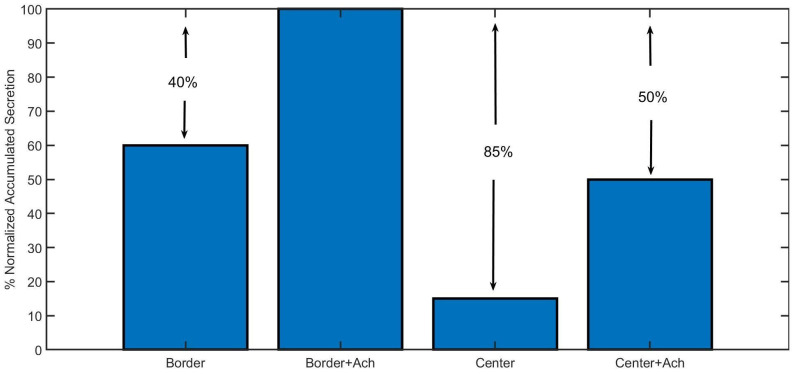
Simulated accumulated secretion in response to a 50 ms voltage pulse alone (first and third bars), or with a previous pulse of ACh (second and fourth bars). Results obtained with the Border and Center configurations of the model.

Figure 7 shows a summary of the results of the accumulated secretion obtained for the two stimulation protocols used in Figure 4 and Figure 5, that is, for a voltage pulse and for an ACh pulse followed by a voltage pulse. These simulations were thought to quantify the influence of a previous activation of nicotinic receptors (with the Ach pulse) in the secretory response, as well as to compare the Border and the Center configurations. 

In Figure 7, the bar graph compares the normalized accumulated secretion in response to the voltage pulse alone or together with the ACh prepulse for both configurations. As can be observed, the secretion with the Center configuration is remarkably lower than the Border one (between 45% and 50%), emphasizing the importance of the spatial localization of all of the molecular elements involved in exocytosis. Moreover, the presence of an ACh prepulse clearly increases the secretion for both configurations (between 35% and 40%), highlighting the importance of the previous activation of nicotinic receptors in local calcium signals leading to the exocytosis of catecholamines.

## 4. Discussion

The nicotinic receptors in bovine chromaffin cells (predominantly heteromeric α3β4 nAChRs and homomeric α7 [13]) are stimulated by the acetylcholine neurotransmitter released by the sympathetic nerves. Specifically, the dysregulation of the α3β4 subtype has been associated with relevant human disorders such as nicotine dependence, cancer, Alzheimer’s and Parkinson’s diseases [2], and insulin resistance [22]. 

Under situations where a person must be ready to survive, catecholamines released by chromaffin cells are very specialized hormones that communicate this vital information to the whole body. Catecholamine secretion is associated with calcium in two ways: one small calcium influx through nicotinic receptors, and one huge influx through calcium channels [6]. The calcium current passing through nicotinic receptors is, in fact, a very small part of the total calcium current (about 2.5%) needed for exocytosis [23], but it seems to be crucial for two reasons: to load vesicles into the active zones of secretion [6], and to directly improve exocytosis, since receptors appear close to the active zones, too [12]. The study of this calcium-regulated process and the spatial coordination of calcium mechanisms is of high importance since the fusion of chromaffin granules contributes to the understanding of neurosecretion [7,18]. Thus, in this work, we were interested in analyzing the contribution of nicotinic receptors to the calcium signals leading to exocytosis in chromaffin cells.

Using fluorescent confocal microscopy, we confirmed that α3β4 nicotinic receptors appear to be mainly located close to the secretory machinery and are associated with the edges of the F-actin cytoskeleton cages inside the cells (Figure 2 and Figure 3). Indeed, we previously reported that they can come even closer after ACh stimulation than after voltage depolarization [12]. Although an interaction between these α3β4 receptors and the α7 subtype in human chromaffin cells has been described [4], we decided to analyze only the α3β4 subtype because we have our own experimental data of these receptors in bovine chromaffin cells, and because they seem to make a more substantial contribution to the secretion induced by ACh [24,25,26]. 

Due to its importance, a large amount of experimental work has focused on the study of the different subtypes of calcium channels and their function in the secretory response of chromaffin cells; see reviews [1,18,19] and the references contained therein. In our computational model, we assume that the VDCC cluster is made of two P/Q-type and one L-type calcium channels, following the reported relative densities for bovine chromaffin cells [1,18], the major contribution of non-L channels to exocytosis [18,19], and taking into account data showing that clusters of these specific channel types are located close to SNARE microdomains [17]. Besides the calcium channels, we also included models for the vesicles and secretory machinery, as well as the nicotinic receptors. This arrangement of subcellular mechanisms allowed us to computationally reproduce the role of the actin cytoskeleton on secretion [10] and the reported calcium influxes through VDCCs leading to exocytosis [1]. 

We observed high local calcium peaks between 8 and 11 μM in the submembrane region, an area with a radius of 0.3 μm (calcium maps in Figure 4C and Figure 6A), in complete agreement with values for local high-calcium microdomains of 300 nm accepted for chromaffin cells [8], which have been reported to trigger vesicle fusion [27]. In contrast, as shown in Figure 4B and Figure 5B,C, the average intracellular calcium was much lower (below 3 μM) and did not exhibit significant differences, while the local calcium was particularly heterogeneous. This contrasting behavior is compatible with the importance of local submembrane calcium domains to regulate exocytosis in chromaffin cells [28].

Nicotinic receptors participate in enhancing the differences between local and average calcium values, as well as calcium distributions. In Figure 4C and Figure 6A, we show calcium maps obtained with the Border configuration that have higher peak values in a more restricted area (tightly coupled active zone) when the receptors are stimulated with an ACh pulse. These results lead to Figure 7, where we quantified the secretory events for both configurations, with or without an ACh pulse preceding a weak depolarization pulse that partially stimulates VDCC opening. Our data indicate that the spatial localization of the molecular elements, along with the stimulation of nicotinic receptors, plays a crucial role in improving the secretory response, supporting the idea that nicotinic receptors promote the co-localization of VDCCs and vesicles to prepare the cell to release a huge volume of catecholamines in case of vital situations, as hypothesized in some works [6,29], and in accordance with the vision of the cytoskeleton as a dynamic structure modulating exocytosis [10,30]. Moreover, it has been reported that mutations in nicotinic receptors could alter their localization and functioning [31], leading, for example, to the dysregulation of the stress response in knockout mice [32].

Our results point out that the calcium signals leading to exocytosis in bovine chromaffin cells are finely modulated by nicotinic receptors since they generate a non-linear calcium entry that, although made of a few ions, increases cell excitability and improves the response to physiological events due to the recognized steep calcium dependence of secretion [33]. The organization of active zones as clusters of calcium channels and receptors close to vesicles, as well as the location of this ensemble in the borders of the cytoskeleton, demonstrates that it is a perfect design of nature inside these neuroendocrine cells to efficiently respond to chemical and electrical stimulation.

## Data Availability

All data used and/or analyzed during the current study are available from the corresponding author upon reasonable request.
